# A Qualitative Approach for Predicting Enhanced Intersystem Crossing in Chromophore-Radical Systems

**DOI:** 10.1021/acs.jpclett.6c00181

**Published:** 2026-03-13

**Authors:** Yash H. Patel, Philip S. Weiss, Ilya D. Dergachev, Claudia E. Avalos

**Affiliations:** Department of Chemistry, 5894 New York University, New York, New York 10003, United States

## Abstract

Enhanced Intersystem Crossing (EISC) is an important mechanism that allows for formally forbidden population transfer from the singlet to triplet manifold in chromophore-radical (C-R) systems. We use first order perturbation theory to estimate the likelihood of EISC in various organic C-R molecules. The first order mixing coefficient κ between the states involved in EISC depends on the difference in pairwise exchange interactions between photoexcited chromophore electrons and the radical. Exchange coupling constants were calculated with the Heisenberg–Dirac–Van Vleck Hamiltonian using excited state wave functions and energies obtained from the CASSCF/QD-NEVPT2 calculations. The predictions derived using this framework are in a good agreement with the available experimental data on EISC observed with transient absorption spectroscopy.

## Graphical abstract

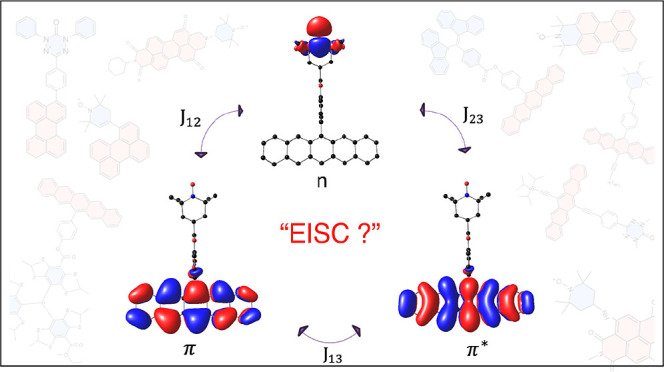

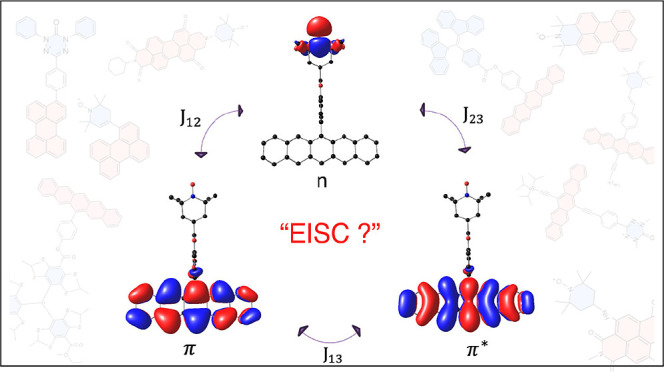

Chromophore-radical (C-R) systems have attracted attention in the spin physics community as potential candidates for dynamic nuclear polarization and quantum sensing due to their favorable optical and spin properties.^1–6^ Similar to the NV^–^ center in diamond, the photoexcitation and subsequent relaxation of a C-R system may lead to a non-Boltzmann ground state spin polarization.^1,7^ In its ground-state configuration, a C-R molecule is characterized by a pair of chromophore-localized electrons in the highest-occupied molecular orbital (HOMO) of π character, and one unpaired electron in a singly occupied molecular orbital (SOMO) localized on a stable radical. The photoexcitation of the C-R leads to the formation of an excited sing-doublet state SD_2_, where an electron from the HOMO orbital is promoted to the lowest unoccupied orbital (LUMO) of the chromophore.^8^ The population from the sing-doublet SD_2_ to the trip-doublet TD_1_ state can be transferred via electron transfer (ET), excitation energy transfer (EET), or enhanced intersystem crossing (EISC).^1,9^ In this paper, we specifically focus on EISC. One mechanism of enhanced intersystem crossing arises from unequal exchange between the two electrons comprising the photoexcited S_1_ state of the chromophore and the radical electron, and it drives the otherwise symmetry-forbidden population transfer from the singlet to the triplet spin manifold in the chromophore on the picosecond time scale.^10^ The relevant spin-coupling interactions involved in the EISC mechanism have been described in previous work.^11^ A general form of the spin-Hamiltonian for a C-R molecule can be defined as:^1^

1 $$\begin{array}{ll} H & =\underset{H_{Z}^{T}}{\underset{︸}{\mu_{B}B_{0}g_{T}{Ŝ}_{T}\;}}+\underset{H_{Z}^{R}}{\underset{︸}{\mu_{B}B_{0}g_{R}{Ŝ}_{R}\;}}\\ & ,+\hbar \left(\underset{H_{J}^{TR}}{\underset{︸}{-2J_{TR}{Ŝ}_{T}\cdot {Ŝ}_{R}}}+\underset{H_{ZFS}^{T}}{\underset{︸}{{Ŝ}_{T}\cdot D_{T}\cdot {Ŝ}_{T}\;}}\right) \end{array}$$

where μ_*B*_ is the Bohr magneton, **B**_0_ is the applied magnetic field, **g** is the *g*-tensor, Ŝ is the spin vector operator, *J*_*TR*_ is the exchange interaction parameter between the chromophore-localized triplet system and the radical-localized doublet system, **D**_**T**_ is the dipolar interaction tensor, and subscripts *T* and *R* refer to the triplet and radical states, respectively. The first two terms on the right-hand side of eq 1 describe the Zeeman effect for the triplet and doublet systems, respectively. The third term describes the spin-exchange between the chromophore and radical electrons *J*_*TR*_, and the fourth term describes the zero-field splitting (ZFS) of the chromophore triplet state, which arises from dipolar interactions between the unpaired electrons. The ratio of the magnitudes of *D*_*T*_ and *J*_*TR*_ loosely defines three exchange-coupling regimes: weak (*J*_*TR*_ ≪ *D*_*T*_), intermediate (*J*_*TR*_ ≈ *D*_*T*_), and strong (*J*_*TR*_ ≫ *D*_*T*_).^10,12^ Previous theoretical studies by Miyokawa et al. employing the complete active space self-consistent field (CASSCF) method predicted that the magnitude of ZFS for the triplet excited states in π-conjugated molecules ranges from 0.01 cm^–1^ to 0.14 cm^–1^, in qualitative agreement with available experimental data.^13^ The magnitude of the triplet/radical exchange parameter *J*_*TR*_ has been predicted (via quasi-degenerate N-electron valence perturbation theory, QD-NEVPT2) to vary by orders of magnitude from fractions to hundreds of cm^–1^ in a series of similar pentacene-based organic C-R systems, and is directly related to the degree of conjugation between the chromophore and radical electrons.^14,15^ A wide range of *J*_*TR*_ values and different coupling regimes are possible, and the ability to reliably predict whether a given C-R system is likely to exhibit EISC based on *ab initio* excited-state coupling calculations would be highly beneficial for informed molecular design.

An accurate description of the open-shell C-R systems requires multireference wave function such as one obtained with the complete active space self-consistent field method (CASSCF).^16^ *J*_*TR*_ and *D*_*T*_ values can be obtained with the use of a multireference perturbation theory such as QD-NEVPT2 or multistate complete active space self-consistent field perturbation theory (MS-CASPT2). Isotropic *J*_*TR*_ values have previously been obtained with the CASSCF/QD-NEVPT2 method in three-electron three-center C-R systems using the formula:^10,12,14^

2 $$E_{TQ_{1}}-E_{TD_{1}}=-\frac{3}{2}J_{\mathrm{TR}}$$

where *TQ*_1_ and *TD*_1_ denote the trip-quartet and trip-doublet states, respectively. The sign of *J*_*TR*_ reflects the ordering of the trip-doublet and trip-quartet states and informs whether the system is antiferromagnetically $\left(E_{TQ_{1}}>E_{TD_{1}}\right)$ or ferromagnetically $\left(E_{TQ_{1}}<E_{TD_{1}}\right)$ coupled.^17^ The Δ*J*_*TR*_ mechanism has been suggested as one possible driving mechanism allowing EISC in C-R systems.^18^

Unequal exchange interaction between an electron occupying the orbital localized on the radical (**n**) (Figure 1) and the two electrons occupying the frontier π, π* orbitals of the chromophore causes mixing between the SD_2_ and *TD*_1_ states.^19^ Using first-order perturbation theory, the mixing parameter between these two states, κ, can be calculated.^11^ Furthermore, this mixing parameter can be directly tied to the probability of observing a transition between the SD_2_ and *TD*_1_ states:

3 $$\psi_{{\mathrm{SD}}_{2}}=\varphi_{{\mathrm{SD}}_{2}}+\kappa \varphi_{TD_{1}}$$

4 $$\psi_{TD_{1}}=\varphi_{TD_{1}}-\kappa \varphi_{{\mathrm{SD}}_{2}}$$

5 $$\kappa =\frac{\left\langle \varphi_{{\mathrm{SD}}_{2}}\left|{Ĥ}_{ex}\right|\varphi_{TD_{1}}\right\rangle }{\Delta E_{TD_{1}/{\mathrm{SD}}_{2}}}=\frac{\sqrt{3}}{2}\frac{\Delta J}{\left(E_{TD_{1}}-E_{{\mathrm{SD}}_{2}}\right)}$$

6 $$\Delta J=\left(J_{12}-J_{23}\right)/2$$

**Figure 1:**
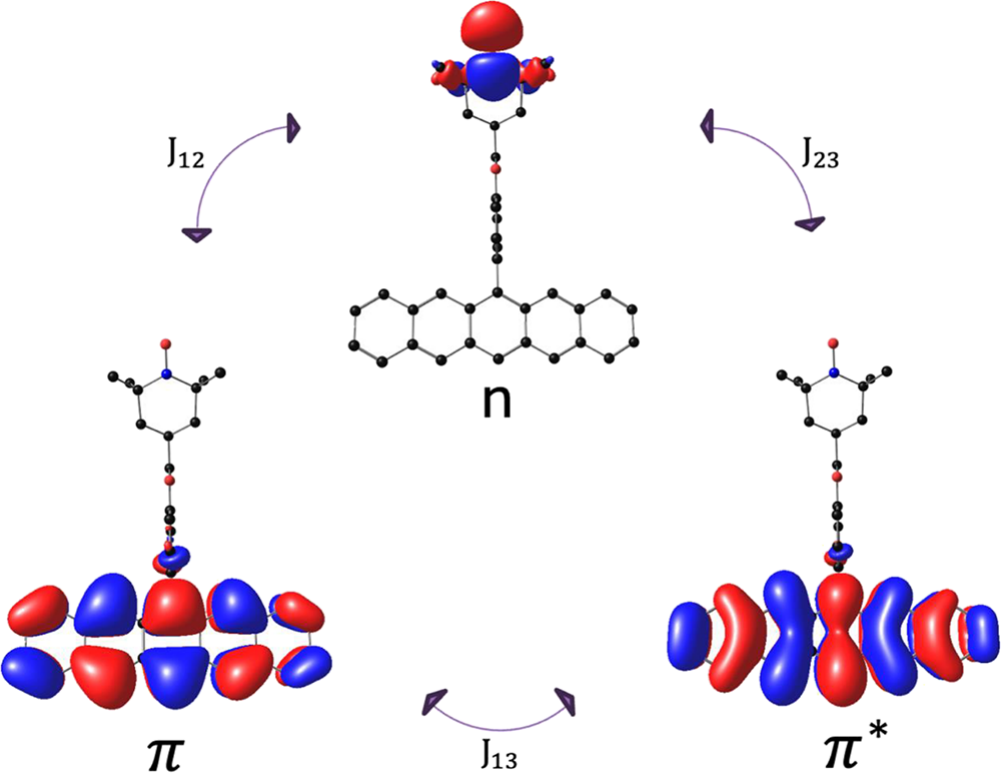
Individual spin-exchange interactions between HOMO π and LUMO π* (*J*_13_), HOMO π and SOMO *n* (*J*_12_), LUMO π* and SOMO n (*J*_23_).

As can be seen in eq 5, the mixing coefficient κ is related to the off-diagonal matrix element that couples the sing-doublet and trip-doublet states. The derivation of κ along with the form of ${Ĥ}_{ex}$ is shown in the Supporting Information.^20^

The parameter κ can be associated with the probability of observing EISC, so we will refer to it as the EISC strength. EISC leads to transitions from SD_2_ to *TD*_1_ on the time scale of 0.1 to 200 ps. It therefore would be beneficial to identify the qualities of C-R molecules which give rise to EISC. It is also important to understand the qualitative ordering of EISC rates for different attachment sites on a chromophore as this will significantly affect κ.

Following the formation of the TD_1_ state, the TQ_1_ state can be populated via ZFS-mediated ISC. This allows for a non-Boltzmann population distribution of the spin sublevels of the TD_1_/TQ_1_ manifold and net polarization of the TD_1_ state.^19,21,22^ However, for ZFS-mediated ISC between the TD_1_ and TQ_1_ states to be effective, one must satisfy specific energy-matching conditions of the TD_1_ and TQ_1_ Zeeman levels as shown in Figure 2(d).^10^ Accurate estimate of a system’s *J*_*TR*_ value then becomes critical for accurate prediction of the external magnetic field strength required to satisfy the matching conditions as shown in Figure 2. In this work, we performed CASSCF/QD-NEVPT2 calculations to obtain the correct electronic wave functions and energies of electronic states in C-R systems. Using these results, we applied first-order perturbation theory to calculate κ between the SD_2_ and *TD*_1_ states.

**Figure 2:**
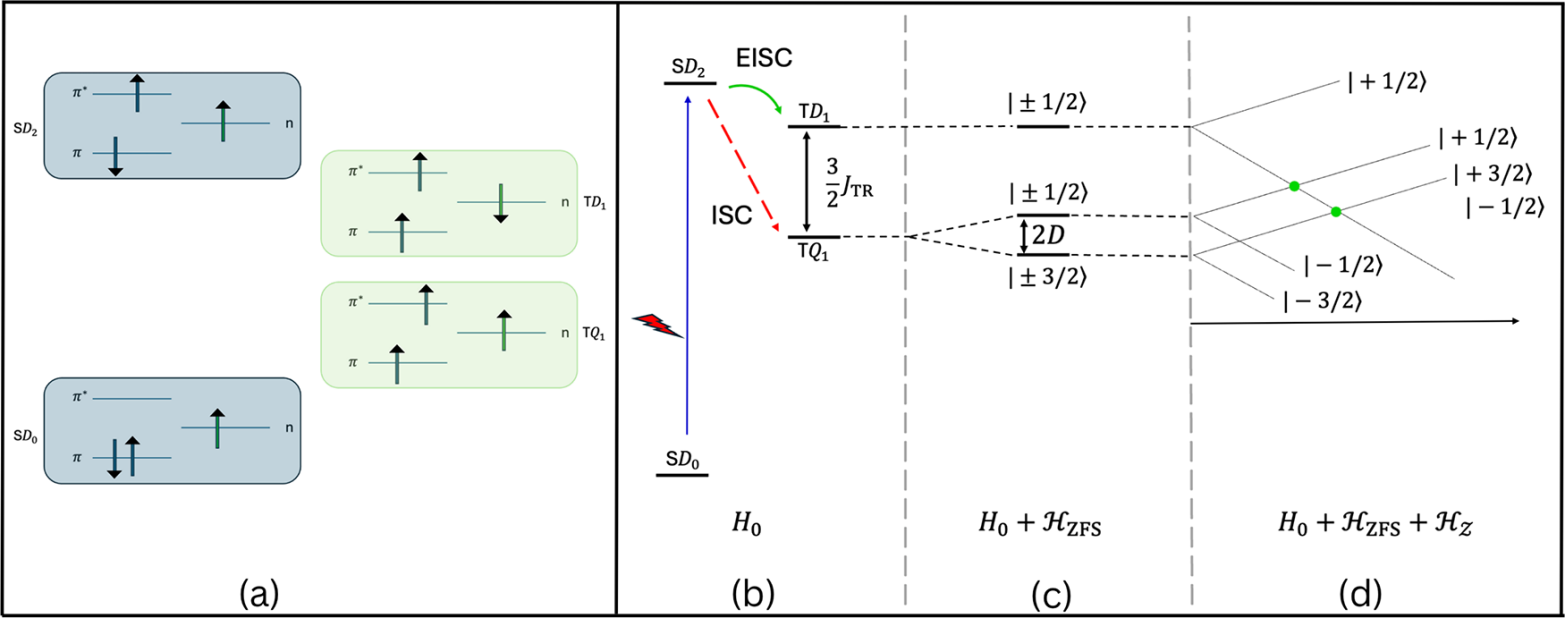
Electron spin configurations of the ground SD_0_, sing-doublet SD_2_, trip-doublet TD_1_, and trip-quartet TQ_1_ states (a), and energy level splitting under (b) zeroth-order (electronic) Hamiltonian H_0_, (c) with addition of the zero-field splitting Hamiltonian H_*ZFS*_, (d) with addition of H_*ZFS*_ and Zeeman Hamiltonian H_*Z*_ showing the crossing conditions between *TD*_1_ and *TQ*_1_ due to the Zeeman splitting.

We predicted the κ parameter for different C-R systems with CASSCF/QD-NEVPT2 calculations. Our work includes the C-R systems previously studied by Weiss et al., and thus we kept the same chromophore and radical labeling indices for the sake of consistency.^14^ They consisted of different sets of chromophores (1*, 2*, ..., 11*) coupled with one of three radicals: 2,2,6,6-tetramethylpiperidine 1-oxyl, TEMPO­(a), α, γ-gbisdiphenylene-β-phenylallyl, BDPA­(b), and Trityl­(c). C-R structures are shown in Figure S4. Due to a large molecular size of the systems involved the trityl radical, the minimal active space composed of three electrons in three orbitals was used and denoted as CAS­(3,3). This active space included the highest occupied molecular orbital (HOMO) and the lowest unnocupied orbital (LUMO) of achromophore and the singly occupied molecular orbital (SOMO) of a radical and can be considered as the “minimal” active space. Figure 3 shows κ values calculated with CAS­(3,3). It shows that the pentacene chromophore attached to a trityl radical tends to exhibit higher κ when compared to TEMPO and BDPA, a prediction that is qualitatively in agreement with the experimental data.^3^

**Figure 3:**
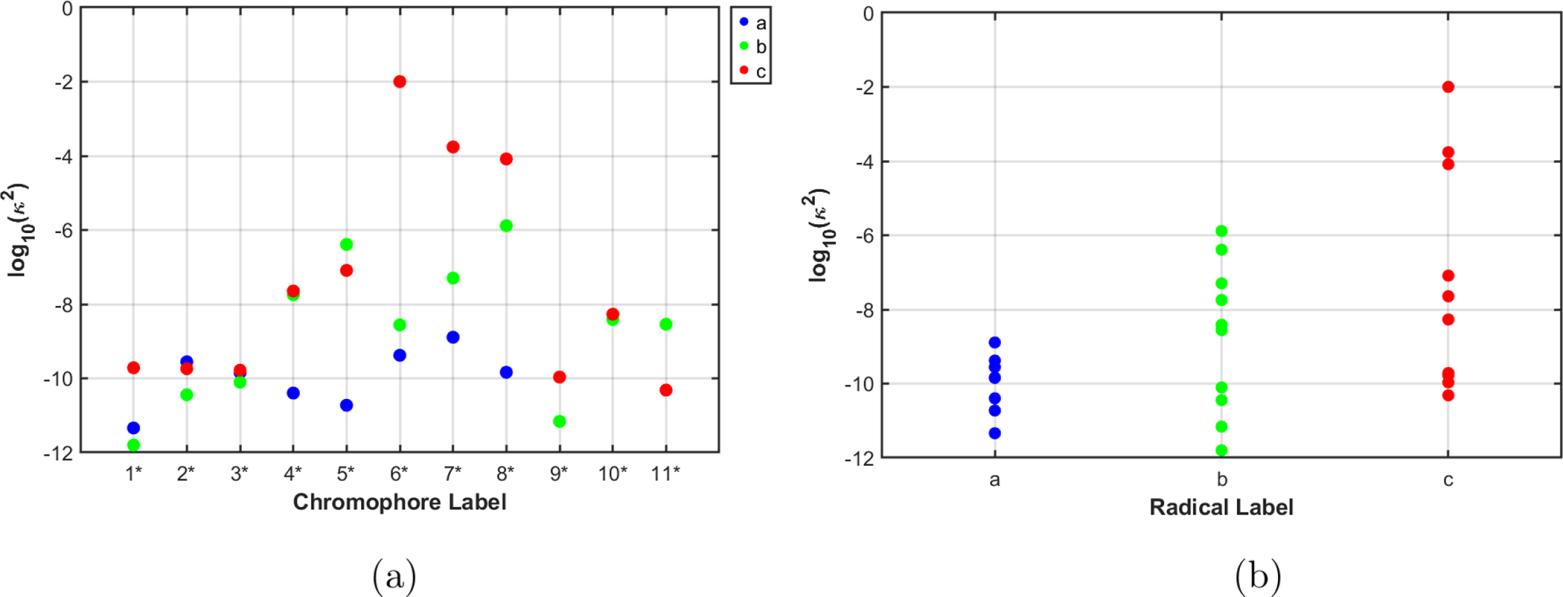
EISC strength in various C-R molecules with respect to (a) specific chromophore for one of the three radicals (blue: TEMPO, green: BDPA, red: Trityl) and (b) with respect to a radical. The corresponding C-R molecules (Figure S4) are shown in the Supporting Information.

In addition to comparing κ between C-R molecules mostly designed in-silico, we also considered various C-R molecules previously studied using femtosecond transient absorption spectroscopy (fs-TA) to allow for comparison to experiment.^3,9,23–29^ EISC is observed as a simultaneous decay of the SD_2_ absorption and corresponding growth in the *TD*_1_ related absorption in the fs-TA spectrum on a picosecond time scale. We calculated κ for previously studied C-R systems using a larger CAS­(5,5) active space. This active space included HOMO–1 and LUMO+1 orbitals of the chromophore in addition to the CAS­(3,3) orbitals. Our analysis provides an approximate minimum value of κ (or log_10_κ^2^ as plotted in Figure 5) above which molecules have been shown experimentally to exhibit EISC, and thus can be used as a powerful predictive tool for a rational design of such systems. The molecules studied in this work are shown in Figure 4 and were previously examined experimentally by means of fs-TA. We note that these results provide a qualitative picture, as κ provides a relative metric to compare the likelihood of a chromophore radical system exhibiting J-mediated EISC. Further details about the approximations made in this approach are described in the Supporting Information.

**Figure 4:**
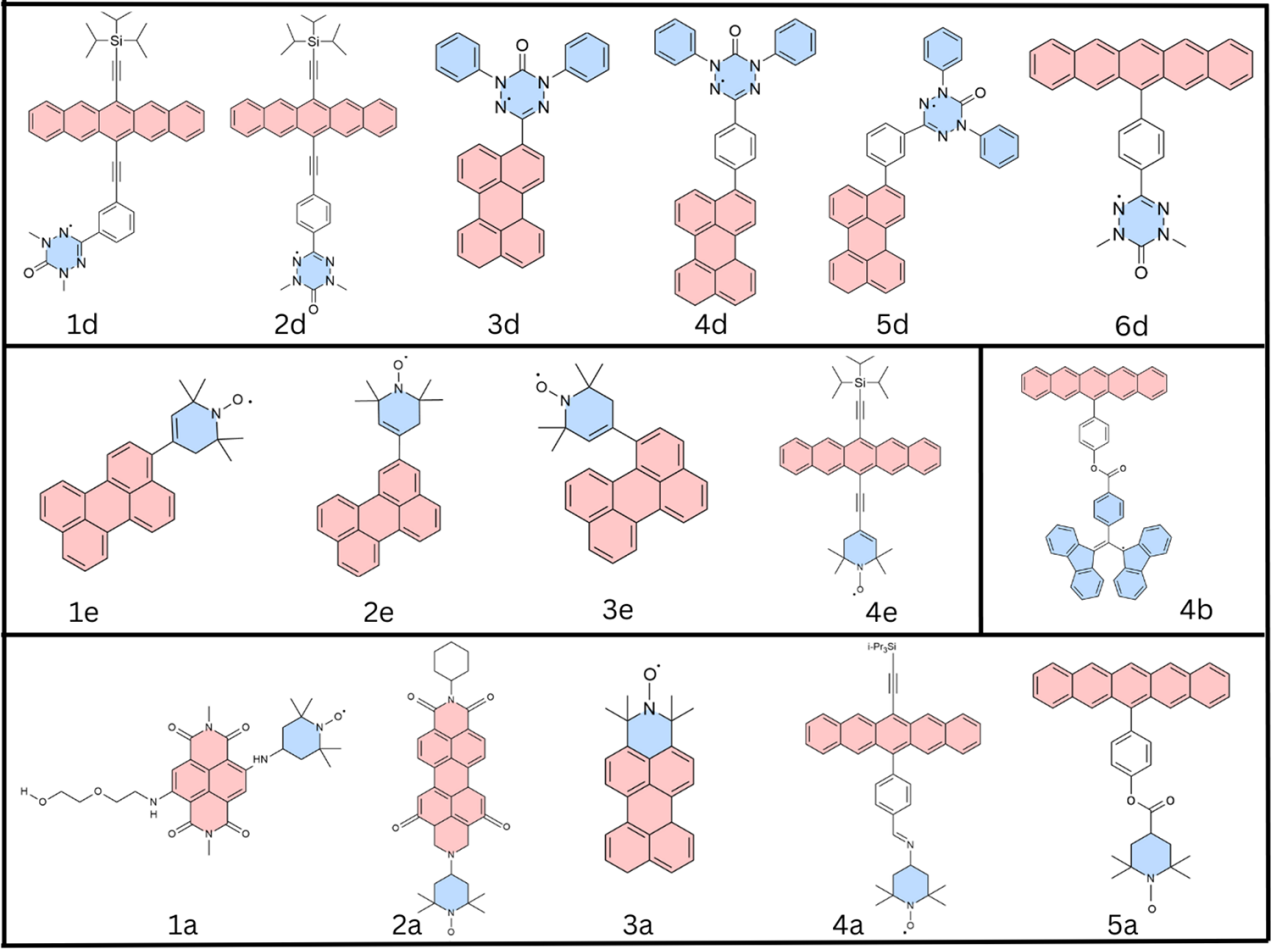
C-R molecules which have been experimentally studied by various research groups: **1d**, **2d**,^23^ **3d**, **4d**, **5d**,^24^ **6d**,^25^ **3a**,^26^ **4b**, **5a**,^3^ **1a**,^27^ **1e**, **2e**, **3e**,^28^ **2a**,^9^ **4a**.^29^

We have observed a correlation between the mixing parameter κ and the probability of observing EISC among the molecules studied. In our analysis of the parameter κ, molecules lying above the red region shown in Figure 5 exhibit EISC from the SD_2_ to the *TD*_1_ state (see also Table 1). We have observed only one exception, molecule **2a**, for which the value of κ is predicted to be too small to exhibit EISC, and contradicts with the experimentally observed fast triplet formation.^30^ To explore the origins of this contradiction, we additionally performed CAS­(7,7) and CAS­(9,9) calculations for **2a**. Because perylenediimide is a larger molecule than perylene or pentacene, we increased the active space to include more orbitals from the π system of perylenediimide. An increased active space provided more accurate excitation energies as shown in Figure S3. Additionally, in order to check for the possibility of additional stable ground-state conformers that may give rise to a κ value encouraging EISC, we conducted a potential energy surface scan along the dihedral angle between the chromophore and the radical in **2a** as shown in Figure S1. However, no additional stable ground-state geometries were located. Details of this analysis are given in the Supporting Information. In our analysis using CAS­(7,7) and CAS­(9,9) methods, the EISC strength approached the red line threshold but did not exceed it. The experimental paper on molecule **2a** provides a detailed discussion of alternative mechanisms, such as electron transfer (ET) and excitation energy transfer (EET), which may contribute to triplet formation in conjunction with EISC.^9^

**Figure 5:**
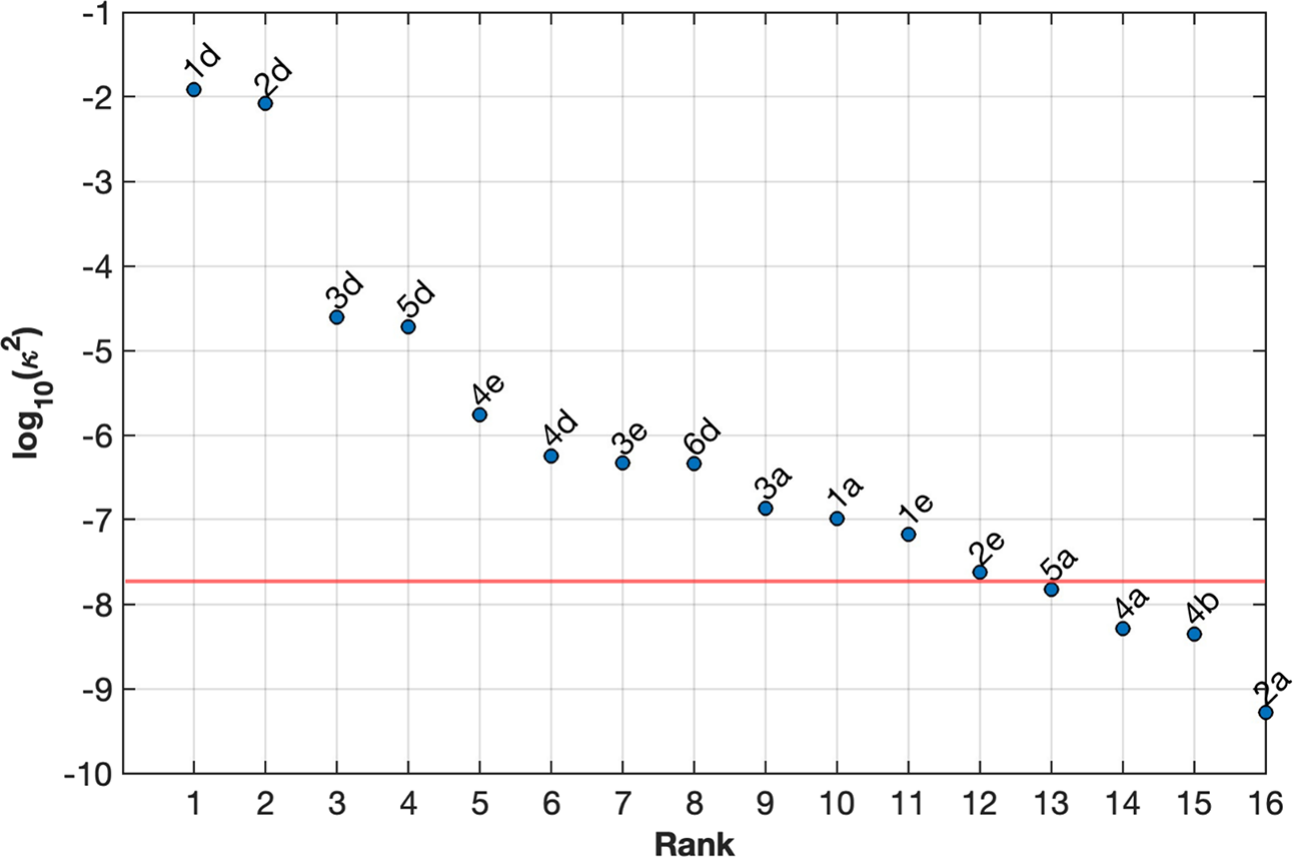
EISC strength of experimentally studied C-R molecules shown in Figure 4. Energies and J-exchange parameters were obtained from the (5,5) CASSCF/QD-NEVPT2 calculations shown in Table 1. Molecules above the red line exhibit EISC.

**Table 1:** Excited State Energies and J-Exchange Parameters Calculated Using (5,5) CASSCF/QD-NEVPT2

Label	TD_1_ (eV)	SD_2_ (eV)	TQ_1_ (eV)	*f*_*osc*_	J_12_ (cm^–1^)	J_23_ (cm^–1^)	J_13_ (cm^–1^)	TD_1_ (cm^–1^)	TQ_1_ (cm^–1^)	J_*TR*_ (cm^–1^)
**2d**	0.937	0.949	0.933	0.12	19.650	28.490	117.577	7557.0	7520.9	24.067
**2a**	1.534	2.149	1.534	1.00	–0.154	–0.040	4964.213	12371.7	12371.8	–0.067
**4d**	1.996	2.383	1.991	0.70	22.329	24.683	3145.390	16096.6	16061.3	23.533
**6d**	1.194	1.050	1.193	0.11	0.995	1.779	–1160.636	9627.8	9625.8	1.333
**4b**	1.302	1.222	1.302	0.13	–0.080	–0.037	–646.023	10501.0	10501.1	–0.067
**5d**	1.998	2.400	2.003	0.69	–17.750	–31.940	3212.871	16115.2	16152.8	–25.067
**3a**	1.773	2.563	1.769	0.93	19.479	21.836	6398.504	14298.2	14267.2	20.667
**3d**	1.862	2.241	1.868	0.75	–43.872	–28.712	3020.202	15014.4	15068.9	–36.333
**1d**	1.002	1.011	1.006	0.17	–19.048	–27.060	55.360	8079.3	8114.1	–23.200
**3e**	2.073	2.679	2.080	0.59	–36.316	–39.650	4852.429	16719.4	16776.4	–38.000
**2e**	1.884	2.688	1.884	0.71	–0.168	0.830	6483.839	15194.3	15193.8	0.333
**1e**	2.002	2.450	2.002	0.60	1.123	0.189	3616.027	16148.2	16147.2	0.667
**1a**	1.836	1.748	1.836	0.30	0.296	0.524	–711.707	14809.7	14809.1	0.400
**4e**	0.968	0.924	0.967	0.15	2.073	2.540	–348.068	7804.7	7801.2	2.333
**5a**	1.255	1.128	1.255	0.12	–0.076	–0.201	–1021.585	10118.8	10119.0	–0.133
**4a**	1.104	1.078	1.104	0.14	–0.162	–0.177	–206.111	8903.2	8903.5	–0.200

In conclusion, we were able to qualitatively reproduce the following experimental observations:

- Molecules **1d** and **2d** exhibit the highest EISC strength among all the previously experimentally studied C-R molecules.
- Correct qualitative ordering of the EISC strength predicted for **1e,** **2e**, and **3e** compared to that observed experimentally by Thielert et al.^28^
- Correct qualitative ordering of the EISC strength predicted for **3d**, **4d**, and **5d** compared to that observed experimentally by Imran et al.^24^

So far, only a few C-R molecules with BDPA and Trityl radicals that exhibit EISC have been reported in the literature. Based on the predicted values of κ, we propose new C-R molecules that are expected to exhibit EISC as shown in Figure 6 (see also Table 2). EISC strength in these structures was predicted to lie above the minimum value for κ (log κ^2^ = −8) shown in Figure 5.

**Figure 6:**
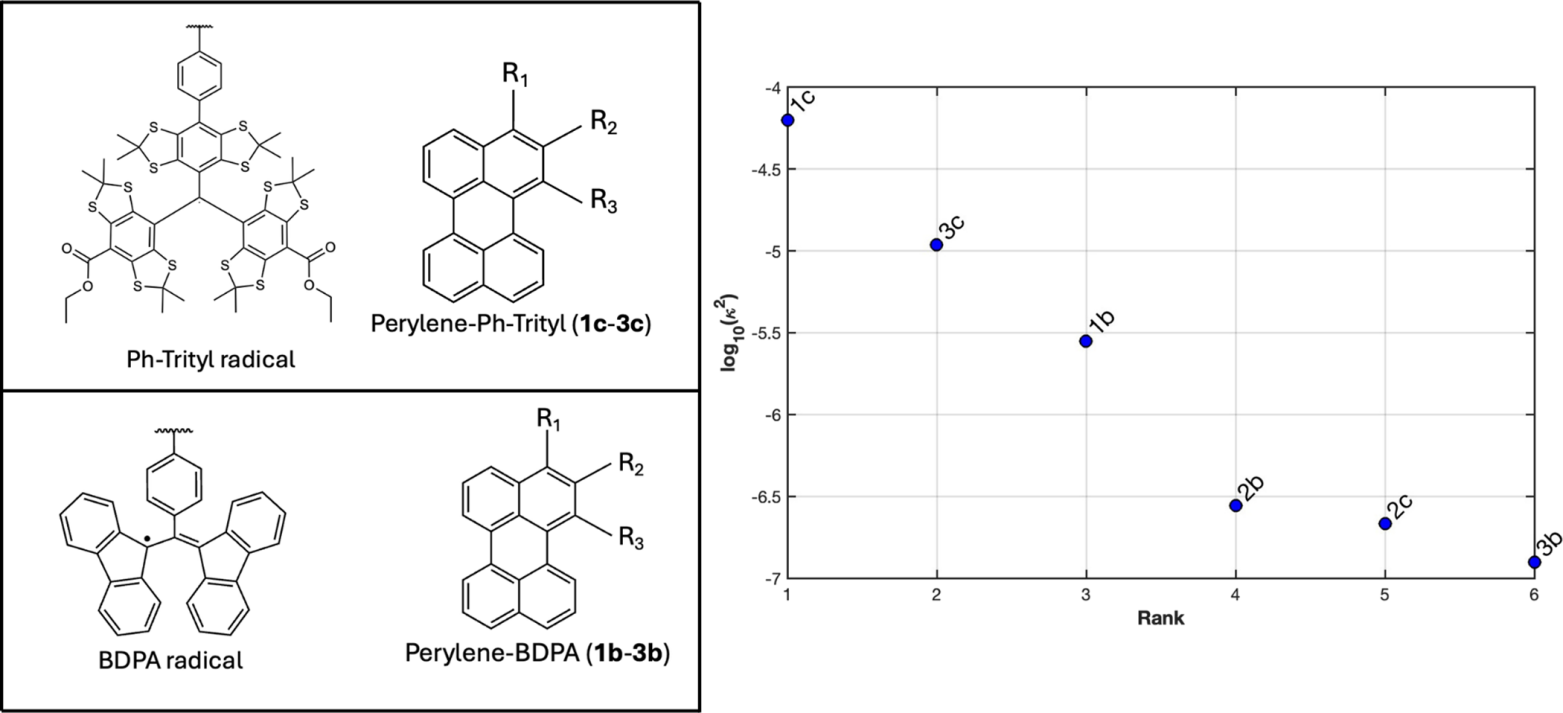
EISC strength of the proposed new structures of C-R molecules. Energies and J-exchange parameters were determined using (5,5) CASSCF/QD-NEVPT2 method as shown in Table 2.

**Table 2:** Excited State Energies and J-Exchange Parameters Calculated Using (5,5) CASSCF/QD-NEVPT2 for Structures Shown in Figure 6

Label	TD_1_ (eV)	SD_2_ (eV)	TQ_1_ (eV)	*f*_*osc*_	J_12_ (cm^–1^)	J_23_ (cm^–1^)	J_13_ (cm^–1^)	TD_1_ (cm^–1^)	TQ_1_ (cm^–1^)	J_*TR*_ (cm^–1^)
**1b**	1.809	2.373	1.805	0.84	15.905	25.445	4573.719	14587.3	14556.3	20.667
**2b**	1.821	2.481	1.821	0.80	0.975	4.629	5322.462	14690.2	14686.0	2.800
**3b**	1.837	2.426	1.833	0.71	24.652	22.358	4773.761	14816.1	14780.9	23.467
**1c**	1.823	2.535	1.826	0.66	15.549	–50.709	5728.211	14702.7	14729.2	–17.667
**2c**	1.821	2.586	1.822	0.71	0.279	–3.767	6166.770	14690.2	14692.8	–1.732
**3c**	1.801	2.502	1.803	0.75	2.337	–23.061	5647.788	14523.0	14538.6	–10.400

## Computational Methods

The geometry optimization was carried out with the unrestricted Kohn–Sham (UKS) density functional theory using the B3LYP exchange-correlation functional and the def2-SVP basis set. The tight SCF convergence criteria (TightSCF) was applied. Multireference electronic structure calculations were carried out using the complete active space self-consistent field (CASSCF) method coupled with the quasi-degenerate N-electron valence perturbation theory (QD-NEVPT2) for dynamic correlation correction to the electronic energies. A resolution of identity auxiliary technique was employed (RI-JK), with *def2/JK* fitting for the Coulomb and exchange integrals and *def2-tzvp/C* fitting for multireference methods. All calculations were performed with the ORCA 6.0.1 software package.^31^ The active space made of three electrons in three orbitals, CAS­(3,3), was used for a preliminary test on a potential EISC strengths in C-R molecules (Figure 3). For a more reliable comparison of the predicted EISC strength and experimental data a larger CAS­(5,5) active space was used for the experimentally studied molecules (Figure 5). For molecule 2a, we report on the advantage of using CAS­(7,7) and CAS­(9,9) as shown in the Supporting Information.^30^

## Supplementary Material



## Notes

Additional details including energy tables, figures, calculation of mixing parameters and results obtained using distinct active spaces (PDF)
