# Supplementary material for: A Qualitative Approach for Predicting Enhanced Intersystem Crossing in Chromophore-Radical Systems
Source: J Phys Chem Lett. 2026 Mar 13;17(12):3515–21. doi: 10.1021/acs.jpclett.6c00181 (PMC13034466; doi:10.1021/acs.jpclett.6c00181)
Supplement: Supplementary file 1 [file jz6c00181_si_001.pdf]

Supplementary Material: A Qualitative Approach for Predicting Enhanced Intersystem Crossing in Chromophore-Radical Systems

Yash H. Patel, Philip S. Weiss, Ilya D. Dergachev, and Claudia E. Avalos*

Department of Chemistry, New York University, New York, NY 10003, United States

E-mail: claudia.avalos@nyu.edu

Contents

1 Perylenediimide-TEMPO 2

2 Chromophore-Radical Structures from[1] 4

3 (3,3) Active Space Calculations of C-R 5

4 Supplementary tables 8

5 Calculation of Mixing Coefficient 8

6 Determining J parameters using CAS(5,5) 11

7 Choice of active space for CAS(5,5) 14

1 Perylenediimide-TEMPO

We calculated the energies of the excited states of PDI-TEMPO as well as the corresponding J-exchange interaction parameters using four different active spaces. The results are shown in table S1. In order to assess differences in the relative energies of these states with changes in the dihedral angle between the chromophore and radical we ran a surface energy scan for this molecule, 2a, as shown in figure S1. The associated κ values obtained for 2a using different active spaces are shown in figure S2. The effect of expanding the active space on the accuracy of the calculated SD_2 excitation energy is shown in terms of absolute error (eV) in figure S3.

Table S1: CASSCF/QD-NEVPT2 excited state energies and J-exchange parameters calculated using (3,3), (5,5), (7,7), and (9,9) active spaces for 2a molecule.

Label	TD_1 (eV)	SD_2 (eV)	TQ_1 (eV)	f_{osc}	J_{12} (cm^{-1})	J_{23} (cm^{-1})	J_{13} (cm^{-1})	TD_1 (cm^{-1})	TQ_1 (cm^{-1})	J_{TR} (cm^{-1})
2a(3,3)	1.484	1.983	1.484	0.83	0.015	-0.063	4022.08	11969.4	11969.4	0
2a(5,5)	1.534	2.149	1.534	1	-0.154	-0.040	5166.496	12305.7	12305.8	-0.066
2a(7,7)	1.709	2.242	1.709	0.87	-0.155	-0.009	4292.654	13786.3	13786.5	-0.133
2a(9,9)	1.718	2.410	2.718	0.78	-0.191	-0.014	5582.74	13858.7	13858.8	-0.067

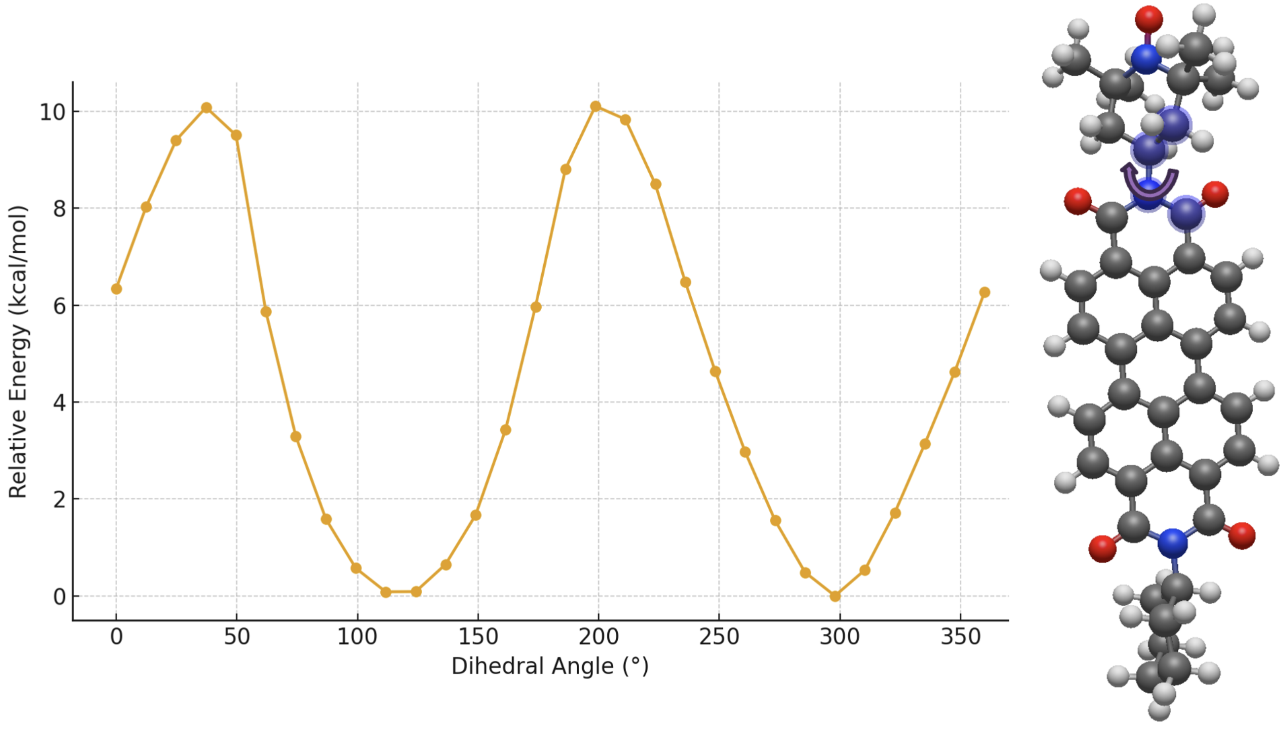

Figure S1: Surface energy scan for molecule 2a as a function of the dihedral angle between the perylenediimide chromophore and TEMPO radical.

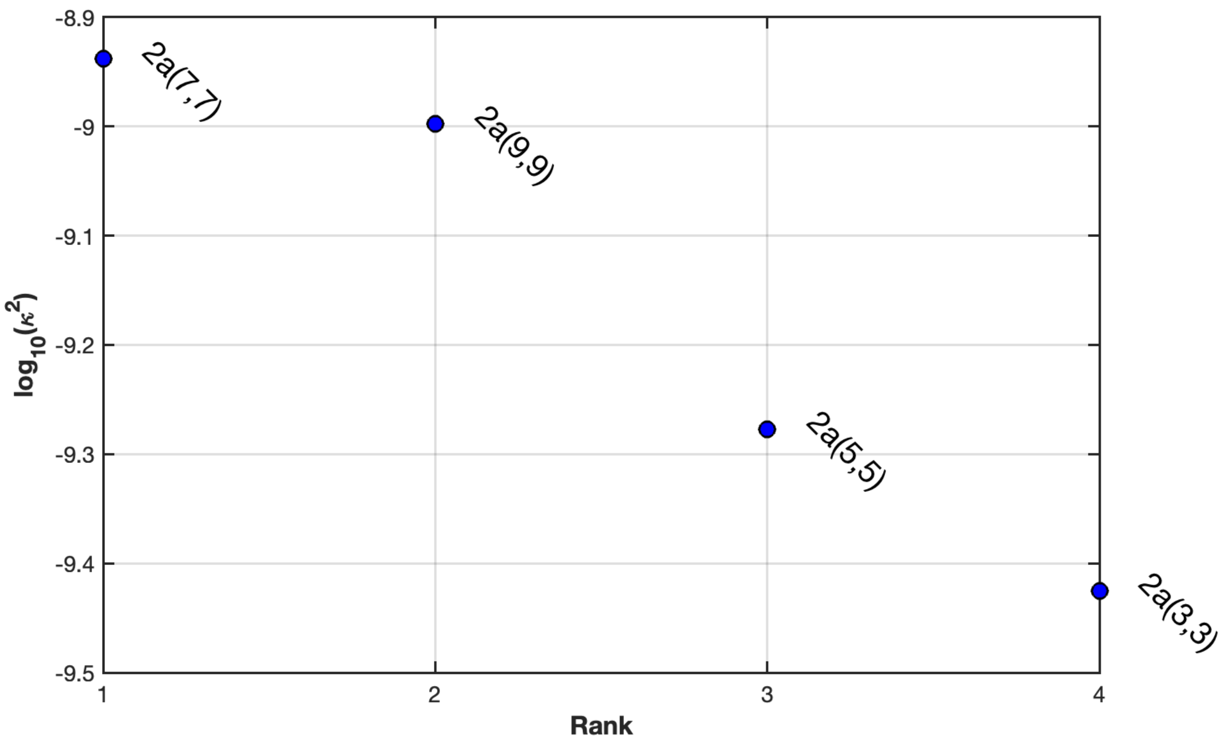

Figure S2: EISC strength predicted for molecule 2a as the function of the active space. From the data shown in Table S1.

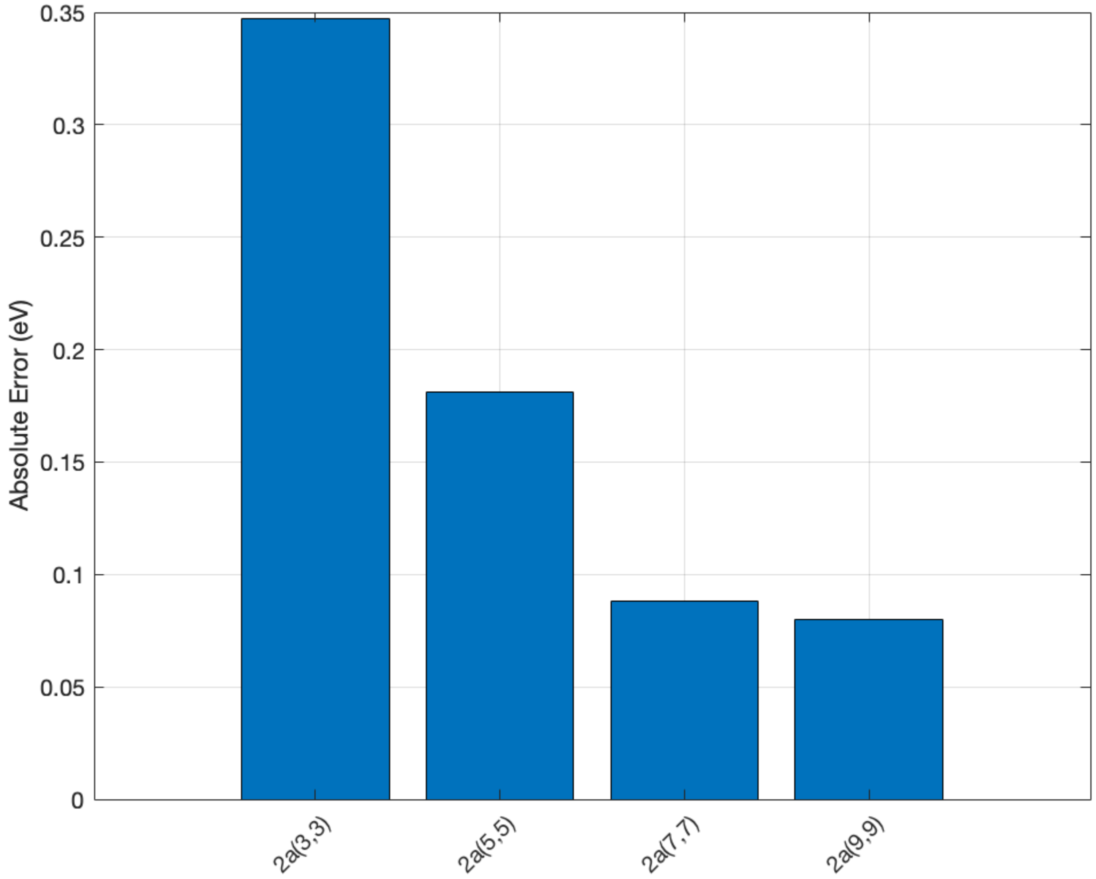

Figure S3: Absolute error computed for the SD_2 excitation energy in molecule 2a for different active space with respect to the experimental value (2.33 eV).[2] From the data shown in Table S1.

2 Chromophore-Radical Structures from[1]

The structures described in reference[1] are shown in figure S4 as a reference for the main text discussion.

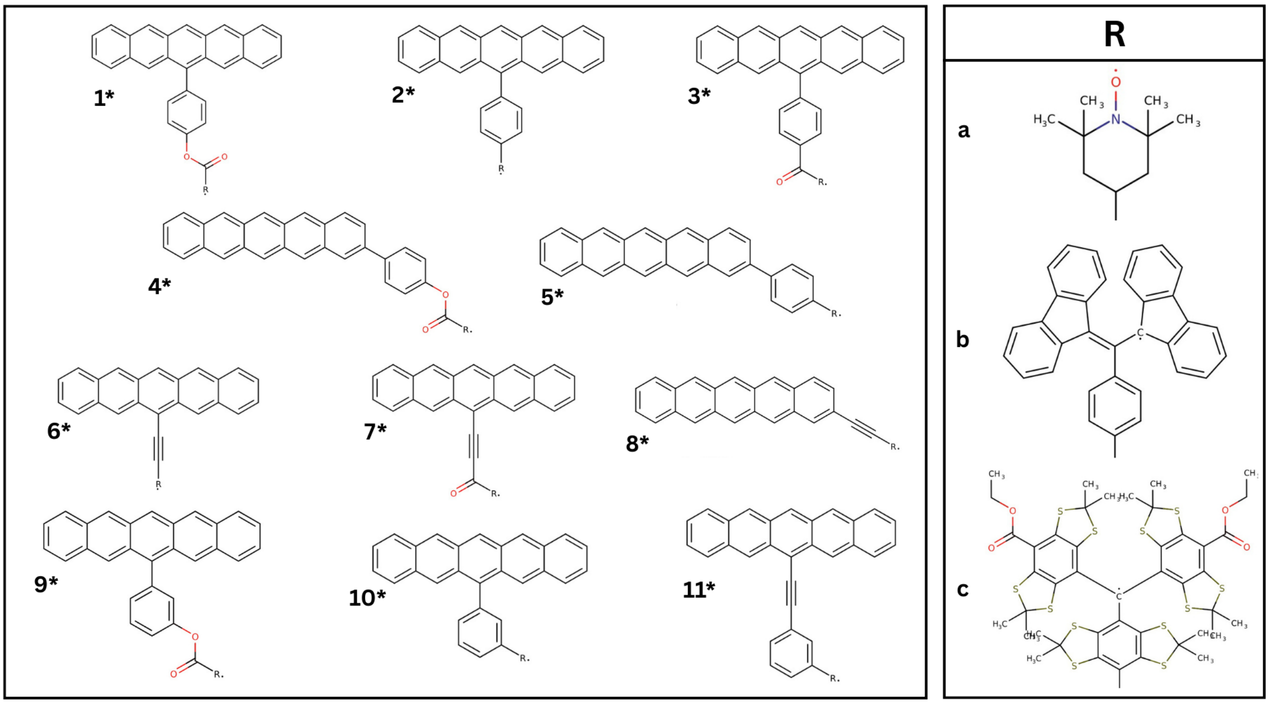

Figure S4: Chromophore-radical molecules investigated in previous work.[13]

3 (3,3) Active Space Calculations of C-R

The parameter κ^2 depends on the $|J_{12} - J_{23}|^2$ value as well as energy difference between excited sing-doublet and trip-doublet state. In order to investigate the variation of the κ^2 value for CAS(3,3) and CAS(5,5). We first plot variation in individual J components for CAS(3,3) and CAS(5,5) as shown in figure S5. From the figure S7, we can observe that molecules 2d,5a and 4a seem to show the highest deviation in terms of κ^2 value.

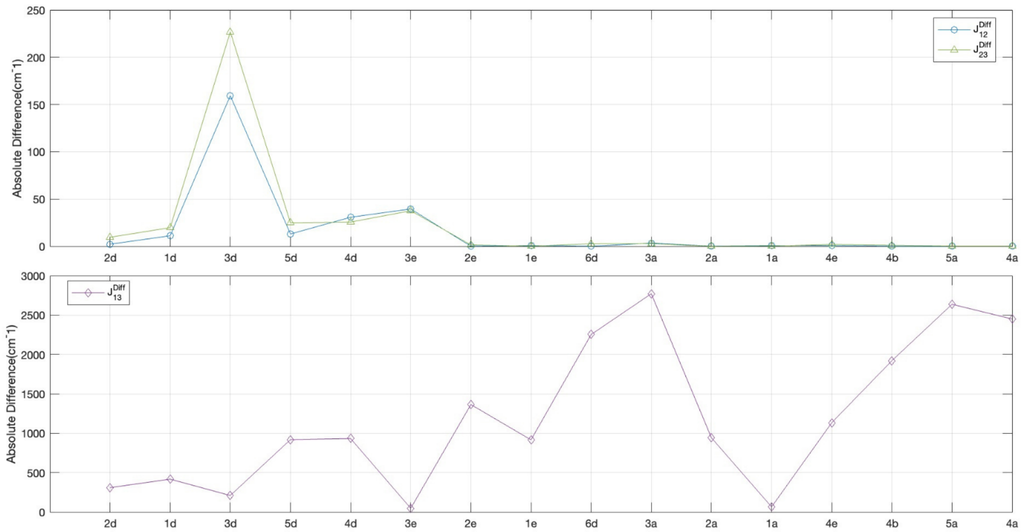

Figure S5: Difference in values of J_{12},J_{23} and J_{13} between CAS(3,3) and CAS(5,5) method.

$$J_{12}^{Diff} = |J_{12}(3,3) - J_{12}(5,5)|,$$
$$J_{23}^{Diff} = |J_{23}(3,3) - J_{23}(5,5)|, \qquad (1)$$
$$J_{13}^{Diff} = |J_{13}(3,3) - J_{13}(5,5)|$$

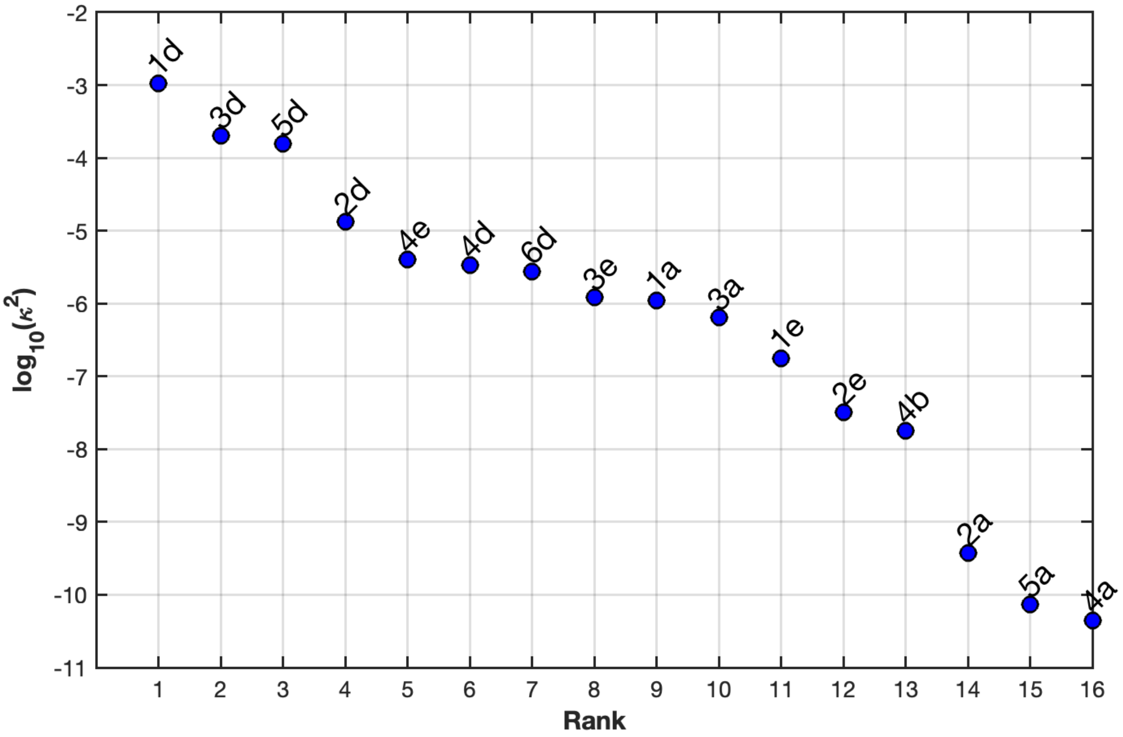

Figure S6: EISC strength predicted from the CASSCF/QD-NEVPT2 calculations with the (3,3) active space for experimental structures shown in Figure 4 of the main text. These κ values are extracted from data shown in Table S2.

The choice of active space can have an effect on the magnitude and sign of J as well as the components of J. The choice of active space to accurately describe both the state energies and magnetic interactions of a system is a persistent challenge in this class of calculations. In our case, we expanded the active space of the chromophore orbitals to better calculate the SD_0 to SD_2 transition as has been seen for other chromophore energy predictions in pentacene.[4] If we compare the kappa values for the 5,5 case and 3,3 case we do see some variation, particularly in 2d, 5a and 4a. However, qualitatively the same trend in κ^2 is observed. This larger deviation appears to be present only in cases where J_{12} and J_{23} are both very small $<0.01\ cm^{-1}$ or in cases where the relative value of J_{13} is comparable to that of J_{12} and J_{23} This apparent systematic variation should warrant future study.

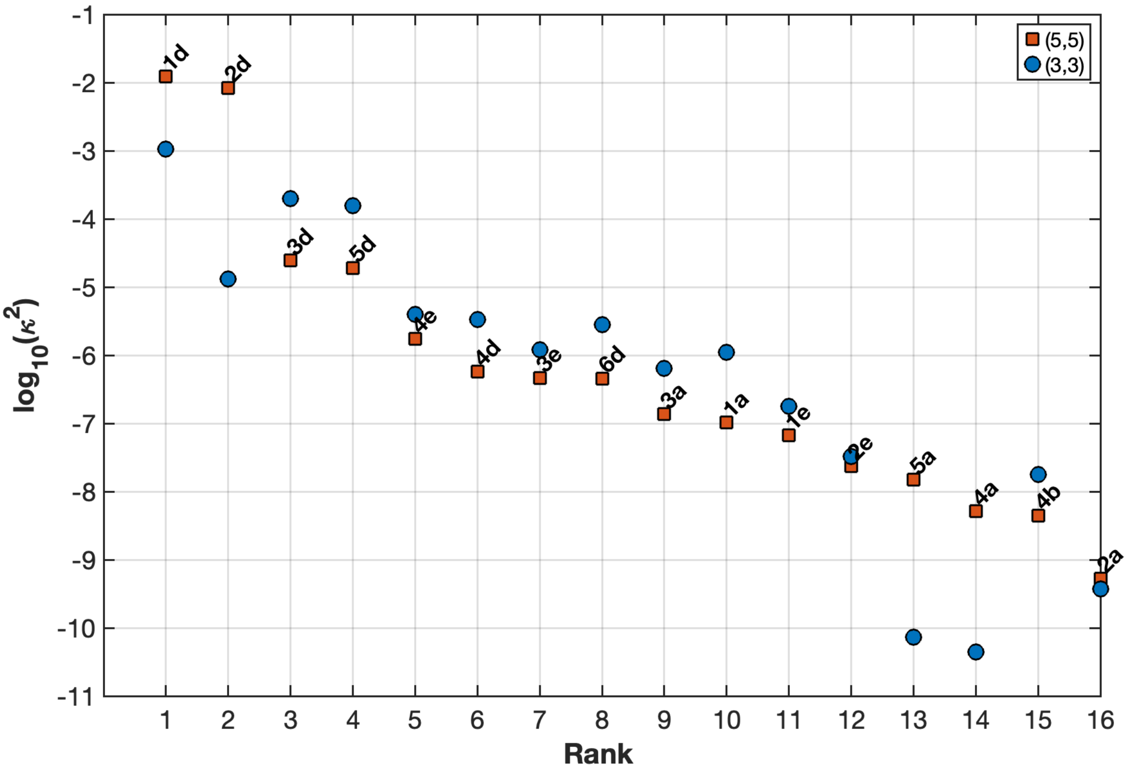

Figure S7: EISC strength comparison predicted from the CASSCF/QD-NEVPT2 calculations with CAS(3,3) and CAS(5,5).

4 Supplementary tables

Table S2: Excited state energies and J-exchange parameters obtained from the CASSCF/QD-NEVPT2 calculations with the (3,3) active space for experimental structures shown in Figure 4 of the main text.

Label	TD_1 (eV)	SD_2 (eV)	TQ_1 (eV)	f_{osc}	J_{12} (cm^{-1})	J_{23} (cm^{-1})	J_{13} (cm^{-1})	TD_1 (cm^{-1})	TQ_1 (cm^{-1})	J_{TR} (cm^{-1})
2d	1.09	1.141	1.087	0.40	17.441	18.932	426.701	8791.1	8763.8	18.200
2a	1.484	1.983	1.484	0.84	0.015	-0.063	4022.08	11969.5	11969.5	0.067
4d	1.803	2.309	1.803	0.85	-8.398	-0.940	4078.731	14515.4	14545	-4.667
6d	1.198	1.334	1.198	0.12	0.975	-0.854	1092.969	9666.2	9666.2	9.000
4b	1.243	1.401	1.243	0.14	0.069	0.101	1275.001	10027	10027	0.000
5d	1.807	2.323	1.813	0.83	-4.732	-56.768	4131.826	14577.3	14623.5	-30.800
3a	1.807	2.254	1.803	0.79	16.074	18.988	3627.44	14573.2	14546	17.533
3d	1.80	2.177	1.843	0.82	-203.142	-255.526	2810.66	14517.6	14861.8	-229.467
1d	1.09	1.153	1.097	0.39	-30.195	-46.771	472.649	8790.9	8848.7	-37.533
3e	1.837	2.432	1.836	0.89	3.200	-2.050	4805.392	14813.1	14812.2	0.600
2e	1.835	2.47	1.835	0.92	-0.256	0.667	5119.341	14802.4	14802.4	0.200
1e	1.801	2.363	1.801	0.97	1.913	0.009	4531.931	14550.9	14526	0.933
1a	1.899	1.803	1.899	0.50	1.065	0.249	-776.741	15317.1	15316.1	6.667
4e	1.051	1.148	1.05	0.14	2.907	4.483	785.229	8478.3	8477.2	3.733
5a	1.287	1.488	1.287	0.24	0.003	0.011	1615.416	10383.5	10383.5	0.000
4a	1.082	1.361	1.082	0.22	0.006	-0.009	2244.159	8729	8729	0.000

5 Calculation of Mixing Coefficient

Here we report the analytical steps to calculate a mixing coefficient between the excited sing-doublet and trip-doublet states. Spin adapted configurations of the excited state wavefunctions are written below. Here a, b and m represent the π, π^* and SOMO orbitals, respectively.

$$\varphi_{\mathrm{SD}_2} = \begin{cases} \dfrac{\left|a\bar{b}m\right\rangle - \left|\bar{a}bm\right\rangle}{\sqrt{2}} \\ \dfrac{\left|a\bar{b}\bar{m}\right\rangle - \left|\bar{a}b\bar{m}\right\rangle}{\sqrt{2}} \end{cases},$$

$$\varphi_{\mathrm{TD}_1} = \begin{cases} \dfrac{2\left|ab\bar{m}\right\rangle - \left|a\bar{b}m\right\rangle - \left|\bar{a}bm\right\rangle}{\sqrt{6}} \\ \dfrac{\left|a\bar{b}\bar{m}\right\rangle + \left|\bar{a}b\bar{m}\right\rangle - 2\left|\bar{a}\bar{b}m\right\rangle}{\sqrt{6}} \end{cases},$$

$$\varphi_{\mathrm{TQ}_1} = \begin{cases} |abm\rangle \\ \dfrac{|ab\bar{m}\rangle + |a\bar{b}m\rangle + |\bar{a}bm\rangle}{\sqrt{3}} \\ \dfrac{|a\bar{b}\bar{m}\rangle + |\bar{a}b\bar{m}\rangle + |\bar{a}\bar{b}m\rangle}{\sqrt{3}} \\ |\bar{a}\bar{b}\bar{m}\rangle \end{cases}.$$

We calculate the mixing coefficient between φ_{SD_2} and φ_{TD_1}:

$$\chi_1 = \frac{|a\bar{b}m\rangle - |\bar{a}bm\rangle}{\sqrt{2}}, \qquad \chi_2 = \frac{|a\bar{b}\bar{m}\rangle - |\bar{a}b\bar{m}\rangle}{\sqrt{2}}$$

$$\eta_1 = \frac{2\,|ab\bar{m}\rangle - |a\bar{b}m\rangle - |\bar{a}bm\rangle}{\sqrt{6}}, \qquad \eta_2 = \frac{|a\bar{b}\bar{m}\rangle + |\bar{a}b\bar{m}\rangle - 2\,|\bar{a}\bar{b}m\rangle}{\sqrt{6}}.$$

Two electron operator is a Coulomb repulsion term between pairs of electrons:

$$\hat{O}_2 = \hat{V} = \sum_{i<j} \frac{1}{r_{ij}}.$$

We calculate all first order mixing coefficients between these two states:

$$\langle \chi_i | \hat{V} | \eta_j \rangle$$

To simplify the calculation steps we use the following two electron operator identities:[5]

$$\hat{O}_2 = \sum_{i<j} \frac{1}{r_{ij}}.$$

Case A: $|K\rangle = |\cdots mn \cdots\rangle$

$$\langle K|\hat{O}_2|K\rangle = \frac{1}{2}\sum_m \sum_n \big([mn|mn] - [mn|nm]\big) = \frac{1}{2}\sum_m \sum_n \langle mn||mn\rangle$$

Case B: $|K\rangle = |\cdots mn \cdots\rangle\,,\ |L\rangle = |\cdots pn \cdots\rangle$

$$\langle K|\hat{O}_2|L\rangle = \sum_n \big([mp|nn] - [mn|np]\big) = \sum_n \langle mn||pn\rangle$$

Case C: $|K\rangle = |\cdots mn \cdots\rangle\,,\ |L\rangle = |\cdots pq \cdots\rangle$

$$\langle K|\hat{O}_2|L\rangle = [mp|nq] - [mq|np] = \langle mn||pq\rangle$$

The non-vanishing coefficients are listed below:

$$\langle \chi_1|\hat{V}|\eta_1\rangle = \frac{1}{\sqrt{2}\,\sqrt{6}}\Big[\underbrace{2\langle a\bar{b}m|\hat{V}|ab\bar{m}\rangle}_{(1)} - \underbrace{\langle a\bar{b}m|\hat{V}|a\bar{b}m\rangle}_{(2)} - \underbrace{\langle a\bar{b}m|\hat{V}|\bar{a}bm\rangle}_{(3)}$$

$$- \underbrace{2\langle \bar{a}bm|\hat{V}|ab\bar{m}\rangle}_{(4)} + \underbrace{\langle \bar{a}bm|\hat{V}|a\bar{b}m\rangle}_{(5)} + \underbrace{\langle \bar{a}bm|\hat{V}|\bar{a}bm\rangle}_{(6)}\Big],$$

$$\langle \chi_1|\hat{V}|\eta_1\rangle = \frac{1}{\sqrt{2}\,\sqrt{6}}\Big[2\underbrace{\langle a\bar{b}m|\hat{V}|ab\bar{m}\rangle}_{K_{bm}\ \text{(case B)}} - 2\underbrace{\langle \bar{a}bm|\hat{V}|ab\bar{m}\rangle}_{K_{am}\ \text{(case B)}}\Big] = \frac{1}{\sqrt{3}}\left(K_{bm} - K_{am}\right),$$

$$\langle \chi_2|\hat{V}|\eta_2\rangle = \frac{1}{\sqrt{2}\,\sqrt{6}}\Big[\underbrace{\langle a\bar{b}\bar{m}|\hat{V}|a\bar{b}\bar{m}\rangle}_{(1)} + \underbrace{\langle a\bar{b}\bar{m}|\hat{V}|\bar{a}b\bar{m}\rangle}_{(2)} - \underbrace{2\langle a\bar{b}\bar{m}|\hat{V}|\bar{a}\bar{b}m\rangle}_{(3)}$$

$$- \underbrace{\langle \bar{a}b\bar{m}|\hat{V}|a\bar{b}\bar{m}\rangle}_{(4)} - \underbrace{\langle \bar{a}b\bar{m}|\hat{V}|\bar{a}b\bar{m}\rangle}_{(5)} + \underbrace{2\langle \bar{a}b\bar{m}|\hat{V}|\bar{a}\bar{b}m\rangle}_{(6)}\Big],$$

$$\langle \chi_2|\hat{V}|\eta_2\rangle = \frac{1}{\sqrt{12}}\Big[-2\langle a\bar{b}\bar{m}|\hat{V}|\bar{a}\bar{b}m\rangle + 2\langle \bar{a}b\bar{m}|\hat{V}|\bar{a}\bar{b}m\rangle\Big]$$
$$= \frac{1}{\sqrt{3}}\left(K_{bm} - K_{am}\right).$$

And the rest are vanishing coefficients:

$$\boxed{\langle \chi_1|\hat{V}|\eta_2\rangle = 0, \qquad \langle \chi_2|\hat{V}|\eta_1\rangle = 0.}$$

6 Determining J parameters using CAS(5,5)

While the CAS(5,5) active space provides a comprehensive description of electron correlation, the resulting wavefunctions contain a mixture of configurations. Python code picks up coefficients of [2duu0], [2udu0], [2uud0] , [2uuu0] from all determinants. These are the primary coefficients that form sing-doublet,trip-doublet and trip-quartet spin states. Our method maps ab initio multi-determinantal wavefunctions onto these relevant localized spin configurations. This is an important step because relevant localized spin configurations are considered to participate in EISC in our analysis. To investigate the validity of our deconvolution method, we do following analysis.

J_{TR} can be calculated in two ways. J_{TR} values in table 1 are given using the formula below, where J_{TR} can be directly extracted using the energy difference between the Trip-Quartet and Trip-Doublet states.

$$E_{TQ_1} - E_{TD_1} = -\frac{3}{2} J_{\mathrm{TR}} \tag{2}$$

We can also extract J_{TR} using J_{12} and J_{23} values with this formula $J_{TR} = 1/2(J_{12} + J_{23})$. Where J_{12} and J_{23} values are extracted by transforming the Hamiltonian from its eigenbasis to determinant basis. Comparing J_{TR} values using both methods gives us validation for individual J_{12} and J_{23} values. Figure S8 compares J_{TR} calculated using both methods for CAS(3,3) and CAS(5,5).

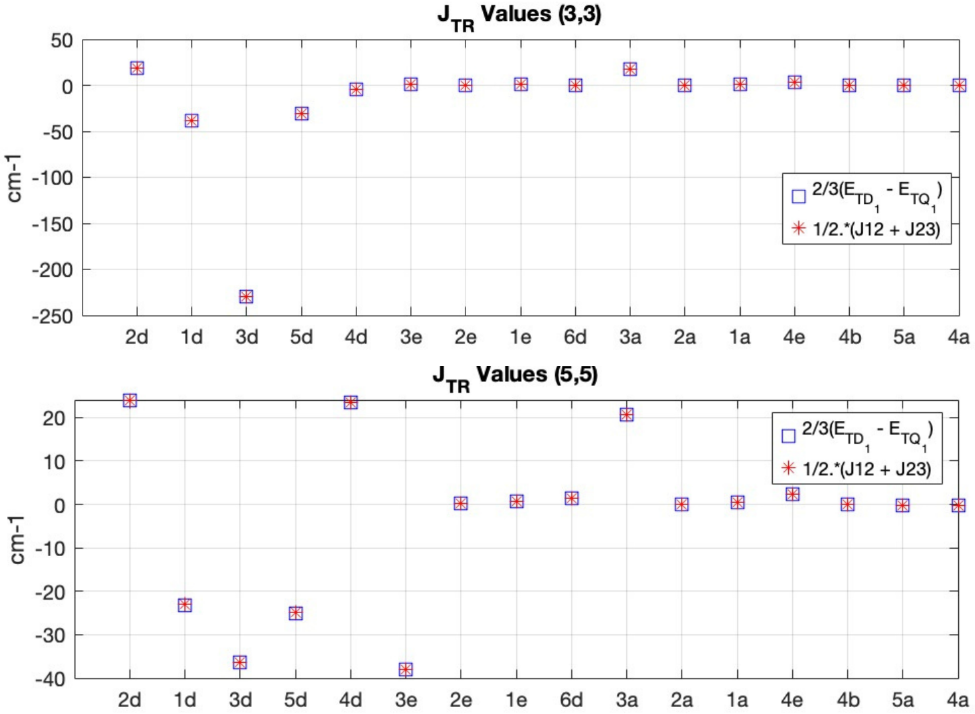

Figure S8

We observed that expanding the protocol for extracting J values from the effective Hamiltonian constructed for the CAS(3,3) active space works well for the CAS(5,5) active space as well. This is because TD_1,SD_2, and TQ_1 states in CAS(5,5) wavefunctions mostly consist of [2duu0], [2udu0], [2uud0] , [2uuu0] determinants, which closely resemble those for the same states in CAS(3,3). We report CASSCF wavefunction coefficient squares for CAS(3,3) and CAS(5,5) determinants in Table S3.

Table S3: Coefficient square comparison for CAS(3,3) and CAS(5,5) CASSCF wavefunctions.

**Label**	TD_1**(3,3)** [duu]	[udu]	[uud]	TD_1**(5,5)** [2duu0]	[2udu0]	[2uud0]
2d	0.1675	0.6665	0.1657	0.1521	0.6084	0.1521
2a	0.1666	0.6665	0.1666	0.1681	0.6561	0.1681
4d	0.1670	0.6663	0.1661	0.1444	0.5929	0.1521
6d	0.1662	0.6665	0.1670	0.1444	0.5929	0.1444
4b	0.1666	0.6665	0.1666	0.1444	0.5776	0.1444
5d	0.1626	0.6597	0.1673	0.1444	0.5929	0.1444
3a	0.1668	0.6665	0.1665	0.1600	0.6561	0.1681
3d	0.1615	0.6642	0.1707	0.1600	0.6241	0.1521
1d	0.1157	0.5712	0.1727	0.1156	0.5929	0.1764
3e	0.1664	0.6655	0.1664	0.1521	0.6084	0.1521
2e	0.1667	0.6665	0.1665	0.1600	0.6241	0.1600
1e	0.1665	0.6660	0.1665	0.1444	0.5929	0.1444
1a	0.1669	0.6655	0.1659	0.1600	0.6241	0.1600
4e	0.1669	0.6657	0.1659	0.1521	0.6084	0.1521
5a	0.1666	0.6665	0.1666	0.1444	0.5929	0.1444
4a	0.1666	0.6665	0.1666	0.1521	0.5929	0.1521

**Label**	SD_2**(3,3)** [duu]	[udu]	[uud]	SD_2**(5,5)** [2duu0]	[2udu0]	[2uud0]
2d	0.4990	0.0000	0.5008	0.4900	0.0000	0.4900
2a	0.4986	0.0000	0.4986	0.4761	0.0000	0.4761
4d	0.4984	0.0000	0.4994	0.4761	0.0000	0.4761
6d	0.4973	0.0000	0.4965	0.4900	0.0000	0.4900
4b	0.4977	0.0000	0.4977	0.4900	0.0000	0.4900
5d	0.4959	0.0000	0.4882	0.4761	0.0000	0.4761
3a	0.4965	0.0000	0.4969	0.4624	0.0000	0.4624
3d	0.5014	0.0000	0.4934	0.4900	0.0000	0.4900
1d	0.4578	0.0005	0.4900	0.5041	0.0000	0.4624
3e	0.4997	0.0000	0.4983	0.4761	0.0000	0.4761
2e	0.4996	0.0000	0.4997	0.4761	0.0000	0.4761
1e	0.4969	0.0000	0.4965	0.4761	0.0000	0.4761
1a	0.4997	0.0000	0.4997	0.4900	0.0000	0.4900
4e	0.4991	0.0000	0.5001	0.4900	0.0000	0.4900
5a	0.4972	0.0000	0.4972	0.4900	0.0000	0.4900
4a	0.4982	0.0000	0.4982	0.4900	0.0000	0.4900

**Label**	TQ_1**(3,3)** [uuu]	TQ_1**(5,5)** [2uuu0]
2d	1	0.9216
2a	1	0.9801
4d	1	0.9025
6d	1	0.8836
4b	1	0.8649
5d	1	0.8836
3a	1	0.9801
3d	1	0.9409
1d	1	0.9216
3e	1	0.9025
2e	1	0.9409
1e	1	0.9025
1a	1	0.9409
4e	1	0.9216
5a	1	0.8836
4a	1	0.9025

7 Choice of active space for CAS(5,5)

CAS(3,3) active space was considered a minimal active space that captures all states of interest. However, a larger CAS(5,5) active space was expected to provide more accurate numerical results due to improved treatment of electron correlation. A similar approach has been applied in our recent paper.[1] Within the CAS(5,5), the choice of active space orbitals is also an important task. For molecules 1d,2d,3d,2a,4d,6d,4b,5d,3a two different solutions in terms of the CAS(5,5) active space compositions were obtained. For the first case in addition to the CAS(3,3) space (HOMO, LUMO, SOMO), one contained HOMO-1 and LUMO+1 orbitals of the chromophore [C, C, R, C, C], and for the second case expanded active space included orbitals associated with the radical and bridge [R, C, R, C, R]. It was interesting then to compare these two cases with the extracted κ^2 values. We observe the same trend in κ^2 for both choices of active space. Figure S10 shows how the choice of active space could affect the EISC strength for these molecules.

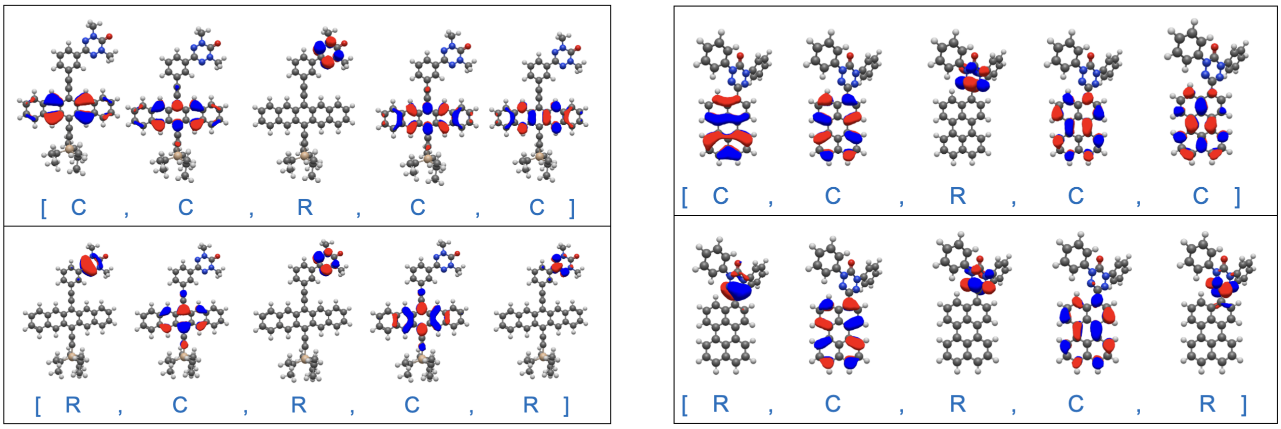

(a) 1d active space orbitals for CAS(5,5)

(b) 3d active space orbitals for CAS(5,5)

Figure S9

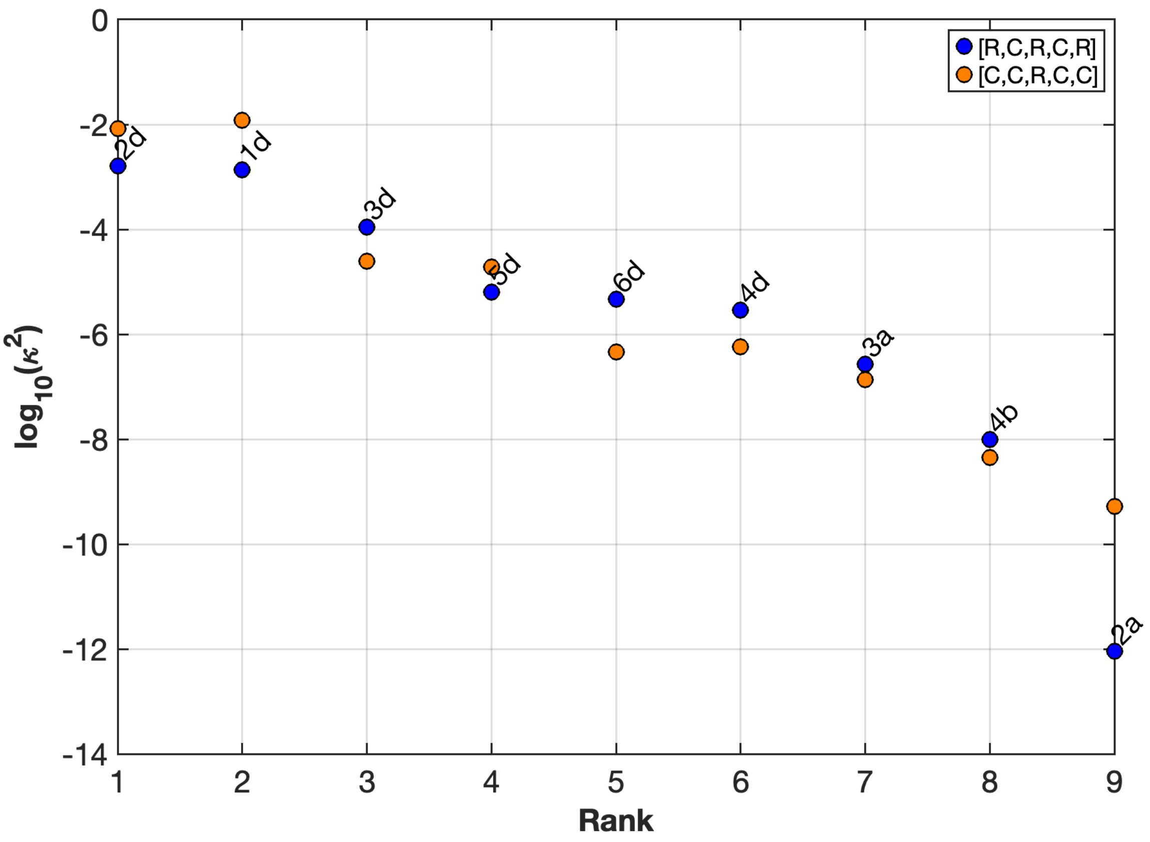

Figure S10: EISC strength comparison predicted from the CASSCF/QD-NEVPT2 calculations with two different active space choices.

References

(1) Weiss, P. S.; Paz, A. S.; Avalos, C. E. An investigation of contributors to the spin exchange interactions in organic pentacene–radical dyads using quasi-degenerate perturbation theory. *Phys. Chem. Chem. Phys.* **2025**, *27*, 8052–8076.

(2) Mayländer, M.; Nolden, O.; Franz, M.; Chen, S.; Bancroft, L.; Qiu, Y.; Wasielewski, M. R.; Gilch, P.; Richert, S. Accessing the triplet state of perylenediimide by radical-enhanced intersystem crossing. *Chemical Science* **2022**, *13*, 6732–6743.

(3) Avalos, C. E.; Richert, S.; Socie, E.; Karthikeyan, G.; Casano, G.; Stevanato, G.; Kubicki, D. J.; Moser, J. E.; Timmel, C. R.; Lelli, M., et al. Enhanced intersystem crossing and transient electron spin polarization in a photoexcited pentacene–trityl radical. *The Journal of Physical Chemistry A* **2020**, *124*, 6068–6075.

(4) Coto, P. B.; Sharifzadeh, S.; Neaton, J. B.; Thoss, M. Low-lying electronic excited states

of pentacene oligomers: A comparative electronic structure study in the context of singlet fission. *Journal of Chemical Theory and Computation* **2015**, *11*, 147–156.

(5) Szabo, A.; Ostlund, N. S. *Modern quantum chemistry: introduction to advanced electronic structure theory*; Courier Corporation, 2012.
